# The energy costs of wading in water

**DOI:** 10.1242/bio.20147831

**Published:** 2014-06-06

**Authors:** Lewis G. Halsey, Christopher J. Tyler, Algis V. Kuliukas

**Affiliations:** 1 University of Roehampton, Holybourne Avenue, London SW15 4JD, UK; 2 University of Western Australia, 35 Stirling Highway, Crawley, WA 6009, Australia

**Keywords:** Bipedal, Metabolic rate, Douglas bag, Wading, Walking, Turning

## Abstract

Studies measuring the energy costs of wading in water have been limited to higher walking speeds in straight lines, in deep water. However, much foraging in water, by both humans and other primates, is conducted in the shallows and at low speeds of locomotion that include elements of turning, as befits searching for cryptic or hidden foods within a patch. The present study brings together data on the rate of oxygen consumption during wading by humans from previous studies, and augments these with new data for wading in shallower depths, with slower and more tortuous walking, to obtain a better understanding both of the absolute costs of wading in typical scenarios of aquatic foraging and of how the cost of wading varies as a function of water depth and speed of locomotion. Previous and present data indicate that, at low speeds, wading has a similar energetic cost to walking on land, particularly at lower water depths, and only at higher speeds is the cost of wading noticeably more expensive than when water is absent. This is probably explained by the relatively small volume of water that must be displaced during locomotion in shallow waters coupled with the compensating support to the limbs that the water affords. The support to the limbs/body provided by water is discussed further, in the context of bipedal locomotion by non-human primates during wading.

## INTRODUCTION

To date there has been a plethora of research examining the costs of walking in humans in entirely terrestrial environments, particularly treadmill-based studies (Halsey and White, 2012). Only a few such studies have been undertaken on more natural terrains (e.g. Pandolf et al., 1976; Pinnington and Dawson, 2001; Zuntz, 1906). Walking in water (wading) to forage is commonplace (Wrangham et al., 2009), and in such cases the energy costs of locomotion could have important ecological consequences because high levels of energy expenditure incurred to displace water while walking would reduce net energy gain. However, only two papers have been published on the energy costs of wading through water. Ghesquiere and Bunkens reported a large increase in rate of energy expenditure in water compared to on land, particularly at higher walking speeds, where the water reached up to the chest (armpits) of the participants (Ghesquiere and Bunkens, 1991). Kuliukas et al. reported data for wading in various depths of water, speeds and with varying knee flexion (Kuliukas et al., 2009). These data are reported as factorial values relative to a single transport cost and thus cannot be easily compared to the values reported by Ghesquiere and Bunkens (Ghesquiere and Bunkens, 1991). Furthermore, as yet there is no information on the costs of wading in water at thigh or knee height.

Humans are not the only primate species known to wade and interestingly, all of the many other primate species observed wading typically exhibit an orthograde (hence bipedal) posture, rising up onto their hind limbs (western lowland gorillas (Doran and McNeilage, 1998), e.g. chimpanzees (Karlowski, 1996), bonobos (Myers Thompson, 2002), several species of monkey (Niemitz, 2010)). Such behaviour is exhibited even when in fairly shallow water, which is likely to be the foraging habitat preference for primates. Niemitz argues that a viewing angle of around 45° improves visual acuity of objects on the riverbed (Niemitz, 2010), and this would not be possible when wading in water close to shoulder height. Furthermore, wading at shallower depths attenuates the cooling effects of water (M. Lieger and C. Niemtiz, unpublished data) while for humans at least, trying to locomote through shoulder-deep water typically triggers swimming behaviour, probably at least in part because of the effort needed to move through the water column due to the volume of water that must be displaced and also the loss of traction on the ground due to the positive buoyancy of the body.

The two previous studies on the energy costs of wading have measured straight line walking, while in reality wading probably tends to consist of (a) small steps as both a response to moving through a more viscous medium and the advantage that slower locomotion affords when searching within foraging patches (Viswanathan et al., 1999), (b) some degree of turning to remain within a foraging patch and respond to cues about food location. Low speeds and also frequent changes in direction associated with foraging will likely serve to increase transport costs (Halsey and White, 2012; Wilson et al., 2013). In the present study we obtained data on the rate of energy expenditure of people wading with short steps and with turns, in water that reached either the knee or the mid-thigh, to validly represent locomotion during typical aquatic foraging, and compared this to the energy costs of the same walking behaviour on land. We also present and consider these data alongside the values reported by Ghesquiere and Bunkens, and Kuliukas et al. (Ghesquiere and Bunkens, 1991; Kuliukas et al., 2009) to provide a fuller picture of how wading costs vary with water depth and walking speed, and how they compare with terrestrial walking costs across the same range of speeds.

## MATERIALS AND METHODS

The Ethics Committee of the University of Roehampton approved this research and written consent was received from each of the participants. Rate of oxygen consumption (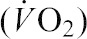) was recorded in participants walking at the same set of slow speeds in control and experimental conditions. The control condition involved walking on land and the experimental condition involved walking in water (wading). For the latter, participants walked around the inside perimeter of a circular paddling pool (2.4 m diameter, 0.8 m high, effective water capacity about 2700 L; Intex Easy Set Pool, Long Beach CA, USA) while for the former they walked inside the perimeter of a set of markers laid out to form a circle of the same diameter as the pool. In each case the participants were therefore slightly turning during each step to maintain an approximately circular path. Furthermore, the participants were asked to do a 180° turn after each completion of the circular path and then continue to walk the path in the opposite direction, thus equalising the total amount of time turning left and turning right during the experiment. Therefore participants included both shallow and acute turns during the experiments.

For the control condition the participants walked at a set of discrete cadences (33, 45, 60 and 90 steps min^−1^), and for each experimental condition also at 75 steps min^−1^, in each case while maintaining the same step length, thus varying walking speed. Cadence was maintained with an auditory metronome. For all conditions, participants were asked to take small steps in order to simulate the sort of walking behaviour most commonly exhibited during wading in water. Specifically, the participants were asked to land the heel of their striding foot only a short way beyond the toes of the standing foot. For each participant, the straight line walking speed of each cadence was calculated such that those data could be compared to previous relevant studies (Ghesquiere and Bunkens, 1991; Kuliukas et al., 2009).

The depth of the water in the experimental condition relative to each participant was measured by recording leg length (as both the height from the ground of the iliac crest, and the height from the ground of the greater trochanter), and immersed leg length. From these data, participants were categorised as either wading in water of knee height (N = 10) or of thigh height (N = 10). Water depth was adjusted when necessary to ensure that for each participant the water either reached the knee cap or approximately the mid-thigh (Table 1). Several participants undertook both experimental conditions. Participant height and weight was also measured.

**Table 1.**
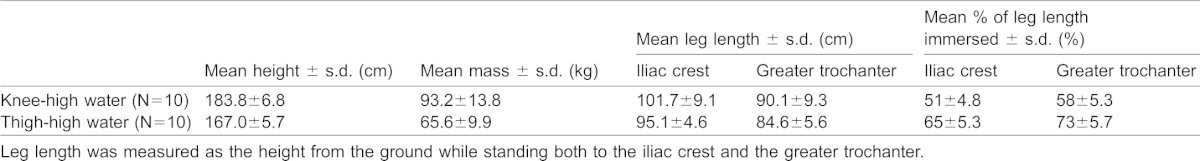
Descriptive statistics of participants in the present study

Oxygen consumption was measured using the Douglas bag method. The Douglas bag was carried on the back of the participant and during the experimental conditions care was taken to ensure that it did not drag in the water. Participants were asked to walk at each cadence for at least two minutes before measurements were taken, to provide sufficient time for cardio-respiratory steady state to be obtained during moderate exercise (Burnley and Jones, 2007; Halsey et al., 2008; Lai et al., 2012; Terrier et al., 2001) and then during a further minute or more, while the participant continued to walk, the expired air was collected in the Douglas bag. Expired air was analysed for oxygen and carbon dioxide concentration (Servomex 1440, Servomex Group Ltd, Crowborough, UK) and volume and temperature (Harvard dry gas meter, Harvard Apparatus Ltd, Kent, England, UK; Fisher Scientific Ltd, Loughborough, England, UK). Surface water temperature was recorded (and varied between 15.4°C and 17.5°C).

### Data compiling

**Fig. 1.**
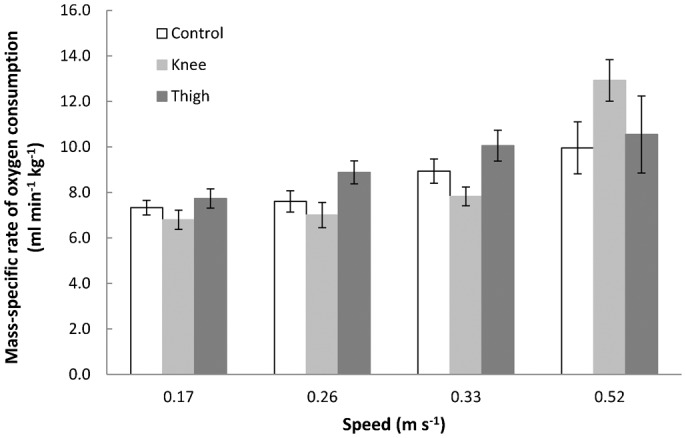
Mean mass-specific rate of oxygen consumption. Mean mass-specific rate of oxygen consumption (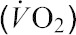) against walking speed for the control condition (walking on land: open bars) and both experimental conditions (wading in knee-high water: light grey bars; and wading in thigh-high water: dark grey bars). Error bars represent ± 1 s.e.m. Data for walking at 0.41 m s^−1^ are not available for the control condition and thus are not presented here for any condition.

Kuliukas et al. reported relative values of 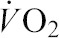 for people wading in a straight line at different speeds (Kuliukas et al., 2009), in comparison to walking on land. These relative values were extracted from figures 1 and 4 of the manuscript using Plot Digitizer (v. 2.6.3). They were converted into estimates of absolute values by benchmarking them against values for walking on land at the same speeds estimated from Halsey and White (Halsey and White, 2012), which provides mean data from a large range of experimental studies on the cost of human pedestrian locomotion, where mean mass is similar to that reported in Kuliukas et al. (76.5 kg) (Kuliukas et al., 2009). Ghesquiere and Bunkens reported absolute values (Ghesquiere and Bunkens, 1991), in figure plots, and thus these were extracted as above. All data are reported as mass specific values in recognition that in the experiments of the present study, water height was defined as relative to the participant's body with shorter participants typically being less heavy. Ghesquiere and Bunkens did not report body mass (Ghesquiere and Bunkens, 1991) and thus based on the participant sample description in that paper, mean body mass is estimated at 65 kg.

## RESULTS

Mass-specific 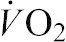 recorded in the present study during the control condition and both experimental conditions are shown in Fig. 1 and Table 2. It can be seen that the effect sizes (the differences in mean mass-specific 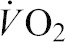 between the conditions at each speed) are typically quite small, particularly between the control condition and the experimental condition where water was at thigh height, although apart from at the highest speed 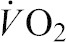 for wading in water to knee height is always slightly lower than for the other two conditions. Subsequent to checking the data for normality and homogeneity of variance, these general observations were confirmed by one-way ANOVAs comparing the three conditions at each of four speeds. All resultant p values were above 0.15 and thus provided little evidence for true differences in mass-specific 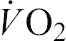 between the conditions at each of the speeds. The standard errors for each condition mean are small (Fig. 1), although larger at the highest speed, suggesting that the sample values are typically fairly accurate estimates.

**Table 2.**
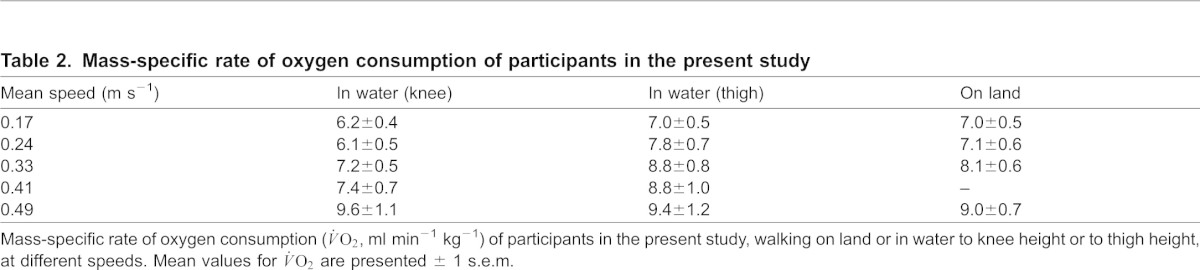
Mass-specific rate of oxygen consumption of participants in the present study

## DISCUSSION

**Fig. 2.**
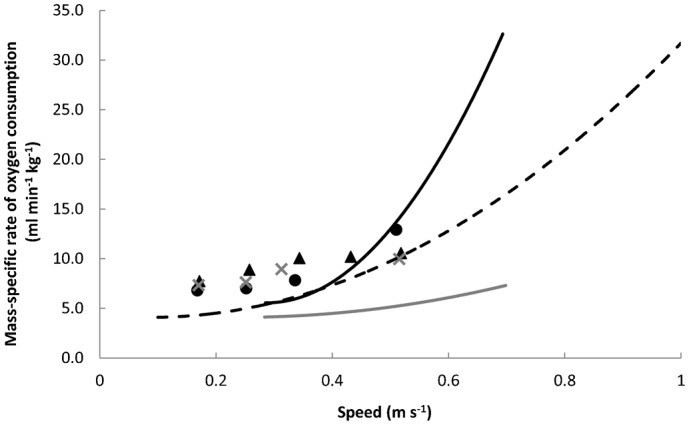
Mass-specific rate of oxygen consumption for current and published data. Mass-specific rate of oxygen consumption (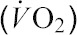) against walking speed for current and published data presented together. Data from the present study are shown as symbols (cross: walking on land, N = 16; triangle: wading in thigh-high water, N = 10; circle: wading in knee-high water, N = 10) while data from previous studies are shown as best fit lines (black: wading in chest-high water (Ghesquiere and Bunkens, 1991); black stippled: wading in waist-high water (Kuliukas et al., 2009); grey: walking on land (Ghesquiere and Bunkens, 1991)). The data from the previous studies indicate an approximate convergence of 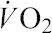 during the different wading scenarios (chest or waist height, or no water) below speeds of around 0.3 m s^−1^. Within the present data, mass-specific 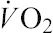 is similar between conditions across the range of speeds tested. Together, the present (see also Fig. 1) and previous data sets therefore suggest that the rate of energy expended during walking on land or wading is similar at lower walking speeds, and the range of speeds during which this is the case is extended upwards when water depth is lower.

Ghesquiere and Bunkens argued that foraging in water is very expensive for humans (Ghesquiere and Bunkens, 1991), who are probably better served in such an environment by horizontal swimming. However, the wading condition in that study involved deep water, reaching up to the armpits of the participants, and was conducted at relatively high locomotion speeds (0.3 to 0.7 m s^−1^; Fig. 2). When wading, humans, and indeed other primates, are likely to prefer shallow water for a number of reasons, including that both food items and predators are easier to see (Niemitz, 2010). Furthermore, heat lost to the water is reduced, and this may well be exacerbated in humans, who appear to have a cranio-caudal division of subcutaneous fat; the majority of subcutaneous fat in both males and females is stored around the lower half of the body, which has been shown via thermal imaging measurements to improve retention of body heat during shallow wading (M. Lieger and C. Niemtiz, unpublished data). The effect sizes in the present study show that typical wading behaviour, i.e. in shallow water (knee- or thigh-high water) and at slow walking speeds, incurs a similar cost of transport to walking on land at the same speeds (Fig. 1). Indeed, people walking in water up to their waists (Kuliukas et al., 2009) and up to their chests (Ghesquiere and Bunkens, 1991) also experience similarly low costs of transport at slower wading speeds; even with such water depths, only at walking speeds above ∼0.35 m s^−1^ is rate of energy expenditure noticeably higher than that on land (Fig. 2). While preferable terrestrial walking speeds for humans average up to around 1.5 m s^−1^ (e.g. Wagnild and Wall-Scheffler, 2013), typical speeds while wading in water, particularly in deeper water, is probably somewhat slower. This is because of the resistance of the water, the reduced traction on the ground due to partial buoyancy, the turning likely inherent in foraging, and the probable reduction in foraging success when wading at a higher pace due to the compromised time to scan any given area for cryptic items (e.g. camouflaged shellfish or partially-buried tubers (Wrangham et al., 2009)) coupled with increased agitation of the substrate and water surface.

Therefore, it might be reasonable to conclude that typical wading by humans during foraging in water up to waist-deep does not incur noteworthy energy costs over and above those incurred during terrestrial foraging. This can most reasonably be explained by the increased costs of moving through water compared to air being relatively small when the water only reaches part way up the lower half of the body, coupled with a possible compensation to that increased cost from the support that the water gives to the body given that the main cost of locomotion is typically the power required to support and propel the body (Gottschall and Kram, 2005).


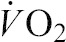
 for a given locomotion speed is somewhat higher in the present study than in both of the previous studies (Fig. 2), even though the wading conditions in the former involved lower water depths. This is perhaps mainly explained by the short step lengths exhibited by the participants to simulate foraging behaviour. Shorter step lengths may result in the feet being in contact with the ground for a shorter duration per step for a given speed, and locomotion costs are thought to increase with the inverse of foot contact time (Kram and Taylor, 1990). An alternative explanation is that shorter steps result in an attenuation of the pendular exchange of kinetic and potential energy exhibited in the normal, striding human gait (Cavagna et al., 1977). However, these increased costs will certainly be in part due to the inclusion of turns in the walking protocol, which incur a significant energetic cost (Wilson et al., 2013). Thus arguably the present data provide more realistic absolute values of immediate energy expenditure for people foraging for cryptic or hidden food, whether in water or on land, and certainly confirm our interpretation of the data from the previous studies that the added cost of walking in water is at most minimal at low speeds.

The support of the limbs by water may have important implications for wading primates beyond humans. Wading in water is considered to provide significant support to a quadrupedal animal in an orthograde posture, even in the shallows (Niemitz, 2010). All non-human primates are obligate quadrupeds and there have been a number of arguments put forward that as such, terrestrial bipedal walking, although possible by these species for short periods of time, may be energetically expensive (Carey and Crompton, 2005; Leonard and Robertson, 2001; Nakatsukasa et al., 2004; Pontzer et al., 2009; Rodman and McHenry, 1980). Although this claim is debated (Kramer and Eck, 2000; Sockol et al., 2007; Taylor and Rowntree, 1973) and only a few studies have directly compared the costs of bipedal and quadrupedal walking in non-human primates, and with mixed conclusions (Nakatsukasa et al., 2004; Sockol et al., 2007; Taylor and Rowntree, 1973), if at least some quadrupedal primates do indeed incur an energy cost during bipedal walking then it is reasonable to suppose that this cost is attenuated while in water due to the support of that medium. Indeed, Kuliukas et al. provide evidence of such, albeit in humans undertaking bent-knee bent-hip walking (Kuliukas et al., 2009). For this reason and others, several recent studies have argued that aquatic wading by hominins could have supported selection for ‘fulltime’ bipedalism (Kuliukas, 2011; Kuliukas, 2013; Kuliukas et al., 2009; Niemitz, 2010; Wrangham et al., 2009). There are a great number of hypotheses proposed to explain the evolution of bipedalism in the hominins, for example Carrier presents an extensive list that includes theories on thermoregulation against ambient heat, carrying, arboreal foraging in the distal branches of trees, defence against predators and aggressive encounters, and male–male physical competition (Carrier, 2011). Some of these theories are more or less based on the argument that humans are relatively energetically efficient at terrestrial locomotion compared to other species (Carrier, 1984; Lieberman and Bramble, 2007; Pontzer et al., 2009; Rodman and McHenry, 1980; Sockol et al., 2007); however, in fact, there is little evidence from metabolic data that humans, or an early hominin *Australopithecus afarensis*, experience(d) low energy expenditure during pedestrian locomotion in comparison to other mammals in general (Halsey and White, 2012; Rubenson et al., 2007). Yet if aquatic wading is invoked as influential in the evolution of bipedalism, an argument can still be made that energy expenditure was an important selection factor. A reduced energy cost of bipedal locomotion while in water for *quadrupedal* hominins (Kuliukas et al., 2009) may well have facilitated the exploitation of aquatic environments, and, as the present study indicates, during such behaviour modern humans do not experience an increased energy cost over equivalent land-based foraging.
